# A machine learning case–control classifier for schizophrenia based on DNA methylation in blood

**DOI:** 10.1038/s41398-021-01496-3

**Published:** 2021-08-03

**Authors:** Chathura J. Gunasekara, Eilis Hannon, Harry MacKay, Cristian Coarfa, Andrew McQuillin, David St. Clair, Jonathan Mill, Robert A. Waterland

**Affiliations:** 1 USDA/ARS Children’s Nutrition Research Center, Department of Pediatrics, Baylor College of Medicine, Houston, TX USA; 2 University of Exeter Medical School, University of Exeter, Exeter, UK; 3 Department of Molecular and Cellular Biology, Baylor College of Medicine, Houston, TX USA; 4 Division of Psychiatry, Faculty of Brain Sciences, University College London, London, UK; 5 The Institute of Medical Sciences, University of Aberdeen, Aberdeen, UK

**Keywords:** Personalized medicine, Schizophrenia

## Abstract

Epigenetic dysregulation is thought to contribute to the etiology of schizophrenia (SZ), but the cell type-specificity of DNA methylation makes population-based epigenetic studies of SZ challenging. To train an SZ case–control classifier based on DNA methylation in blood, therefore, we focused on human genomic regions of systemic interindividual epigenetic variation (CoRSIVs), a subset of which are represented on the Illumina Human Methylation 450K (HM450) array. HM450 DNA methylation data on whole blood of 414 SZ cases and 433 non-psychiatric controls were used as training data for a classification algorithm with built-in feature selection, sparse partial least squares discriminate analysis (SPLS-DA); application of SPLS-DA to HM450 data has not been previously reported. Using the first two SPLS-DA dimensions we calculated a “risk distance” to identify individuals with the highest probability of SZ. The model was then evaluated on an independent HM450 data set on 353 SZ cases and 322 non-psychiatric controls. Our CoRSIV-based model classified 303 individuals as cases with a positive predictive value (PPV) of 80%, far surpassing the performance of a model based on polygenic risk score (PRS). Importantly, risk distance (based on CoRSIV methylation) was not associated with medication use, arguing against reverse causality. Risk distance and PRS were positively correlated (Pearson *r* = 0.28, *P* = 1.28 × 10^−12^), and mediational analysis suggested that genetic effects on SZ are partially mediated by altered methylation at CoRSIVs. Our results indicate two innate dimensions of SZ risk: one based on genetic, and the other on systemic epigenetic variants.

## Introduction

Schizophrenia (SZ), a neurodevelopmental disorder affecting 1% of the world’s population, is characterized by hallucinations, delusion, and cognitive deficits [1]. Although twin and family studies estimate a high heritability for SZ, around 80% [2], the concordance rate of SZ in monozygotic twins is only 50% [3, 4], and genetic variants identified in multiple large genome-wide association studies (GWAS) [5, 6] explain only a small proportion of SZ risk [7, 8]. Although additive effects of these variants enabled the development of a polygenic risk score (PRS) to quantify genetic predisposition for SZ [1, 7], a classifier for SZ case–control status based on PRS performed poorly (area under the receiver operating characteristic curve, AUROC = 0.58–0.70) [9]. Together, these observations led to speculation that, in addition to genetic and environmental factors, epigenetic mechanisms may play an important role in the etiology of SZ [3]. Given the ability of environmental stimuli to affect stochastic developmental epigenetic processes [10–12], epigenetic mechanisms could both explain monozygotic twin discordance and mediate a variety of early environmental risk factors for SZ [13].

Epigenetic regulation involves concerted interactions among various molecular alterations (histone modifications, autoregulatory DNA-binding proteins, etc.). Epigenetic epidemiology, however, focuses almost exclusively on the methylation of CpG dinucleotides in DNA because of its long-term stability and simplicity as a “readout” of chromatin state. Unlike genetic epidemiology, epigenetic epidemiology is complicated by the cell type-specificity of DNA methylation and the potential for reverse causality [14, 15]. Epigenetic variation in peripheral blood may not provide information about epigenetic regulation in the brain [16–18]. Also, in epigenetic epidemiologic studies of SZ, DNA methylation differences between patients and healthy individuals may be a consequence of SZ (e.g., due to medication, increased smoking, etc.) [19]. Genomic regions of systemic interindividual epigenetic variation (SIV) are stable epigenetic polymorphisms established during early development, providing opportunities to overcome these obstacles [15]. By focusing on SIV regions, investigators can use genomic DNA from easily obtainable tissues like peripheral blood to draw inferences about epigenetic regulation throughout the body, including the brain. We recently reported the largest unbiased screen for correlated regions of systemic interindividual epigenetic variation (CoRSIVs) in the human genome [15, 20]. CoRSIVs were identified by analyzing deep whole-genome bisulfite-sequencing (WGBS) data on tissues representing all three germ layers (thyroid, heart, and brain) from each of ten donors from the NIH Genotype-Tissue Expression (GTEx) project [21]. Each of the 9926 CoRSIVs identified is statistically significant (*P* < 0.05), includes at least 5 CpGs, and exhibits an interindividual methylation range of at least 20% [20]. About 50% of CoRSIV-associated genes are implicated in nervous system diseases or mental disorders [20].

Regarding analytical approaches, epigenetic studies of DNA methylation have mainly utilized *t*-tests or other univariate regression methodologies, and focused on detecting associations rather than making predictions [22, 23]. But univariate approaches ignore interactions among features, potentially missing crucial synergistic biological effects [24], motivating increased interest in using machine learning to analyze DNA methylation [22, 25]. A recent machine learning-based method [26] identified an epigenetic signature of SZ in blood DNA using Illumina Human Methylation 450K (HM450) case–control data sets [27]. That approach, however, trains independent machine-learning models focusing on CpGs in biological pathways implicated by gene ontology (GO) analysis, and thus is constrained within existing ontologies. Also, the goal was the identification of differentially methylated positions (DMPs) between cases and controls, not an individualized assessment of SZ risk. Another machine-learning study based on HM450 data on post-mortem brain tissues used a simple decision tree-based algorithm, but detected no significant signals distinguishing cases from controls [28]. Most recently, a poly-methylome score calculated using the DMPs from Hannon et al. (whole-blood SZ case–control DMPs) [27], likewise failed to substantially distinguish cases and controls [29].

Here, we applied two key innovations. First, we focused on the subset of HM450 probes that overlap human CoRSIVs [15, 20]. Second, we used a supervised machine-learning algorithm called sparse partial least squares discriminate analysis (SPLS-DA). Although SPLS-DA has been applied to transcriptomic, metabolomic, and microbiome data [30–32], we are not aware of previous reports applying it to DNA methylation data. Exploiting the regularization (i.e., variable selection) and dimension reduction capabilities of SPLS-DA, in which SZ case–control data can be visualized in a reduced 2-dimension space, we devised a “risk distance” enabling successful identification of a subset of individuals with the greatest risk of SZ. When tested on an independent HM450 case–control data set, our algorithm classified ~85% of SZ cases with a positive predictive value (PPV) of 80%, greatly outperforming a model similarly trained on PRS.

## Materials and methods

### Data

Publicly available Illumina HM450 data sets from five cohorts were used for model training, testing, and validation. The numbers of subjects and availability of additional clinical variables are summarized in Table 1, and demographic characteristics are stated in Supplementary Methods. The training data set (GSE84727, Aberdeen cohort) comprises 414 patients with SZ and 433 non-psychiatric controls who have self-identified as born in British Isles (95% in Scotland) [33]. The model was tested on an independent SZ case–control data set (GSE80417, London cohort) including 353 patients with SZ and 322 non-psychiatric controls born in UK [34]. For additional validation and evaluation of reverse causality by medication use and smoking, the following HM450 data sets were downloaded from NCBI GEO: GSE74193 (prefrontal cortex (PFC) from 191 SZ cases and 335 controls) [35], GSE59685 (whole blood, prefrontal cortex (PFC), entorhinal cortex (EC), superior temporal gyrus (STG), cerebellum (CE) from 67 controls) [36], and GSE50660 (whole blood from 464 smoking and non-smoking individuals) [37].

**Table 1:** Overview of the data sets used in these analyses.

Cohort (GEO accession)	Tissue	Status (phenotype)	Number of samples	PRS available	Antipsychotic use available
Training Data (GSE84727) [33]	Whole Blood	SZ case	414	Yes	Yes
		Controls	433		
Testing Data (GSE80417) [34]	Whole Blood	SZ case	353	Yes	Yes
		Controls	322		
Validation (GSE74193) [35]	PFC (brain)	SZ case	191	No	
		Controls	335		
Validation (GSE59685) [36]	Whole blood	Controls	67		
	EC (brain)				
	PFC (brain)				
	STG (brain)				
	CER (brain)				
Validation (GSE50660) [37]	Whole blood	Smoker	285		
		Non-smoker	179		

For details about the data sets, see Supplementary Methods.

*PFC* prefrontal cortex, *EC* entorhinal cortex, *STG* superior temporal gyrus, *CER* cerebellum, *PRS* Polygenic Risk Score.

### CoRSIV Probes

Previous studies [20, 38, 39] identified human genomic regions (CoRSIVs) that show systemic DNA methylation across diverse tissues of the body. Of the ~480,000 probes on the HM450 array, only 3590 overlap 1982 known CoRSIVs [15]. Because CpG sites within each CoRSIV are correlated, in most analyses we averaged multiple probes within each CoRSIV, yielding 1982 variables. Probes at which blood methylation is known to be correlated with smoking [27] were excluded before training our models.

### Training an SPLS-DA machine-learning model

We identified SPLS-DA as a potentially effective machine-learning method due to its simultaneous variable selection and dimension reduction capability [40, 41]. SPLS-DA operates under the assumption that a small fraction of the original variables is driving the underlying process and uses least absolute shrinkage and selection operator *(LASSO)* regularization [42] for variable selection, shrinking coefficients of unrelated variables to zero. We used the mixOmics R package [43] for implementation (Supplementary Fig. S1).

### Calculating risk distance for training and testing Data

Based on case–control separation in the 2-dimensional (2-D) representation of training samples, a vector can be identified as ${\mathrm{var}}\left( {{\mathrm{dim}}1} \right){{i}} + {\mathrm{var}}\left( {{\mathrm{dim}}2} \right){{j}}$ (Supplementary Methods). Along this vector, Euclidian distance from the origin (0,0) to all training data points can be calculated. Then, for each sample in the independent testing set, 2-D coordinates can be calculated using the same SPLS-DA model parameters identified in the training data, and risk distance computed.

### Model performance evaluation

Model performance is evaluated by setting cutoffs at various risk distance standard deviation multiples (1, 1.5, 2, 2.5, and 3) above the control mean risk distance in the training data, to classify individuals as SZ cases. Positive predictive value (PPV) is the probability that subjects with a positive screening test truly have the disease. To compare the accuracy of different models, we calculated the number of individuals classified as cases at a PPV of 80%, which is considered clinically actionable [44].

## Results

### At CoRSIV probes, DNA methylation is generally positively correlated between blood and brain

**Fig. 1:**
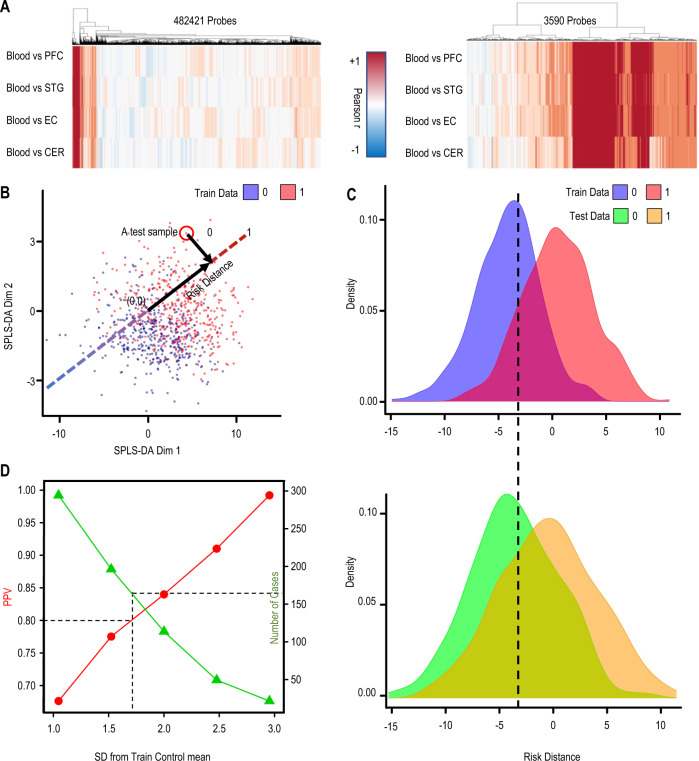
Classification of SZ cases and controls using CoRSIV methylation in blood DNA. **A** Only a small fraction of HM450 probes show a positive correlation of methylation across blood and four brain regions (left). CoRSIVs, however, (right) generally show positive correlations between methylation in blood and these same brain regions. **B** Applying SPLS-DA to CoRSIV data achieved partial separation of cases (1) and controls (0). Euclidean distance (risk distance) from (0,0) to each sample in the 2D plot is calculated along the vector ${\mathrm{var}}\left( {{\mathrm{dim1}}} \right)i + {\mathrm{var}}\left( {{\mathrm{dim2}}} \right)j$. **C** The risk distance distributions for cases and controls in the training data (top). Those for an independent set of cases and controls (testing data, bottom) show similar separation. **D** Evaluation of classifier performance using positive predictive value (PPV). Individuals with risk distance more than (1, 1.5, 2, 2.5, 3) standard deviations (SD) above the mean control risk distance of the training data were considered as positive. The number of individuals at each SD increment classified as cases is shown in green, and PPV is shown in red. By interpolation, a cutoff of 1.7 SD achieves 80% PPV in classifying test cases. By comparison, only 43 out of 307 test controls (14%) pass this threshold.

Our focus on CoRSIVs is based on the rationale that, at these regions, methylation measurements in blood yield information about epigenetic regulation in the brain. Using GSE59685 (blood and four brain regions from each of 67 control individuals) [36], we evaluated Pearson correlations between blood and brain. Only a small subset of HM450 probes consistently showed a strong positive correlation between blood and the four brain regions (Fig. 1A, left). Conversely, at most of the 3590 HM450 probes within genomic regions previously shown to exhibit systemic interindividual epigenetic variation (CoRSIV probes) [20, 38, 39] (Supplementary Table S1) DNA methylation in the blood is, as expected, positively correlated with that in each of the four brain regions (Fig. 1A, right).

### Applying SPLS-DA to classify SZ cases and controls

Using the case–control training data on CoRSIV methylation, we initially attempted to use the tSNE unsupervised machine-learning algorithm to distinguish SZ cases from controls, but there was no separation (Supplementary Fig. S2). Applying SPLS-DA to these same training data, however, partially separated cases and controls into two overlapping clusters (Fig. 1B; the dotted line shows the risk distance vector). SZ cases generally have positive risk distances, and controls tend to have negative values (Supplementary Methods). Importantly, the risk distance distributions of cases and controls show clear separation not only in the training data (Fig. 1C, top) but also in the independent test set (Fig. 1C, bottom). Discrimination of cases and controls improves with increasing risk distance. For example, at the target PPV of 80%, our initial SPLS-DA model based on CoRSIVs classifies 161 of the 353 individuals in the test set as cases (Fig. 1D and Table 2). An SPLS-DA model trained with the same data but with case–control status randomized did not classify a single individual in the test set as a case with 80% PPV (Table 2).

**Table 2:** Comparison of model performance when using different HM450 probe sets.

HM450 probes in the model	SD multiple cutoff for 80% PPV in training set	Cases predicted by algorithm in testing set	% cases predicted in testing set (out of 353 cases)
CoRSIV probes	1.7	161	45%
CoRSIV probes, top range_2–98%_ probes	1.5	278	78%
CoRSIV probes, top range_2–98%_ probes, blood cell composition estimates	1.5	270	76%
Re-trained model (excluded top 10 smoking CpGs and smoking score)	1	253	72%
CoRSIV probes, top range_2–98%_ probes, blood cell composition estimate, smoking (final model)	1	303	85%
PRS	1.3	115	32%
Final model (with PRS)	1	306	86%
Smoking score	1	85	24%
Top variance probes	2	78	22%
Hannon et al.—DMPs detected in blood SZ case–control [27]	1	15	4%
Null model	NA	0	0%

All models were trained on GSE84727 (SZ case–control whole blood) and predicted on test data (GSE80417 SZ case–control whole blood).

### Risk distance is not associated with medication use

**Fig. 2:**
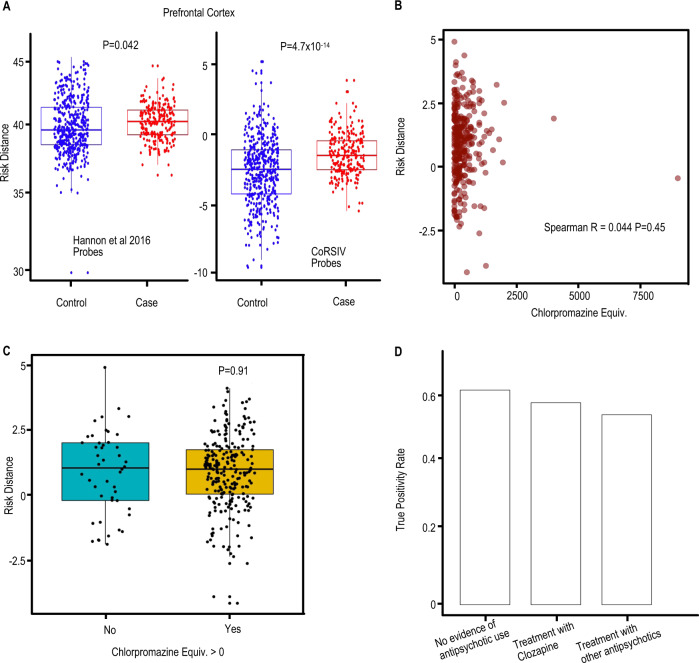
Evidence against reverse causality due to medication use. **A** (Left) Applying a model built on Hannon et al. 2016 probes (SZ case–control DMPs) from blood-based training data to case–control methylation data on the prefrontal cortex (PFC) yields very high-risk distances for cases and controls. By comparison, applying our CoRSIV model trained on blood-based data to the same PFC data set (right) yielded risk distances close to zero and greater separation of cases and controls. **B** For cases in the training data set (Aberdeen cohort; 232 cases with complete drug usage information) risk distances determined by our model are not correlated with chlorpromazine equivalent dose of antipsychotic medication (*P* = 0.45). **C** For this same data set, two classes of cases based on chlorpromazine equivalent dose > 0 (i.e., currently taking medication, *n* = 242) and = 0 (not currently taking medication, *n* = 46) show no difference in mean risk distance determined by our model (*P* > 0.9). **D** In the testing data set (UCL cohort), cases with some use of clozapine (*n* = 60) or other antipsychotics (*n* = 92) were compared with those who have no record of antipsychotic use (*n* = 202). The proportion of individuals correctly classified as cases, based on risk distance, did not differ between groups (*P* > 0.77, *P* > 0.49, odds ratio).

A major caveat is the potential that our findings reflect reverse causality, particularly through an effect of antipsychotic medication. Because antipsychotic drugs can affect the methylation profile in blood by altering the proportion of different leukocyte subtypes [45, 46], it is unlikely that they will induce the same methylation changes in the brain. To test this, we first considered SZ case–control DMPs identified by Hannon et al. [27] as independent variables, and trained an SPLS-DA model using blood DNA methylation data (GSE84727: whole blood from SZ case–control) (Supplementary Fig. S3). Applying this model to methylation data on PFC (GSE74193: whole blood from SZ case–control) [35] yielded very high-risk distances that differed only modestly between cases and controls (*P* = 0.046) (Fig. 2A, left). Applying our CoRSIV-based model to the PFC data, however, yielded risk distances that were close to zero and substantially higher in cases than controls (*P* = 4 × 10^−14^) (Fig. 2A, right), in agreement with our results in blood.

To directly evaluate the effect of medication use on risk distance, we used clinical data from the OPCRIT database [47]. In the training set, there was no association between risk distance and chlorpromazine equivalent dose (*R* = 0.04, *P* = 0.45) (Fig. 2B), and the average risk distance of cases with chlorpromazine equivalent doses > 0 did not differ from that of those not currently on antipsychotic drugs (*P* = 0.9) (Fig. 2C). In the testing set, the proportion of cases correctly classified as such (based on risk distance) was unaffected by the use of antipsychotic drugs (*P* = 0.77 and *P* = 0.49 for treatment with clozapine and treatment with other drugs respectively, relative to no antipsychotic drugs) (Fig. 2D). Together, these data indicate that our classifier is not detecting blood DNA methylation changes induced by the use of psychiatric medications, providing strong evidence against reverse causality.

### Focusing on SIV is crucial to the success of a blood-based classifier

**Fig. 3:**
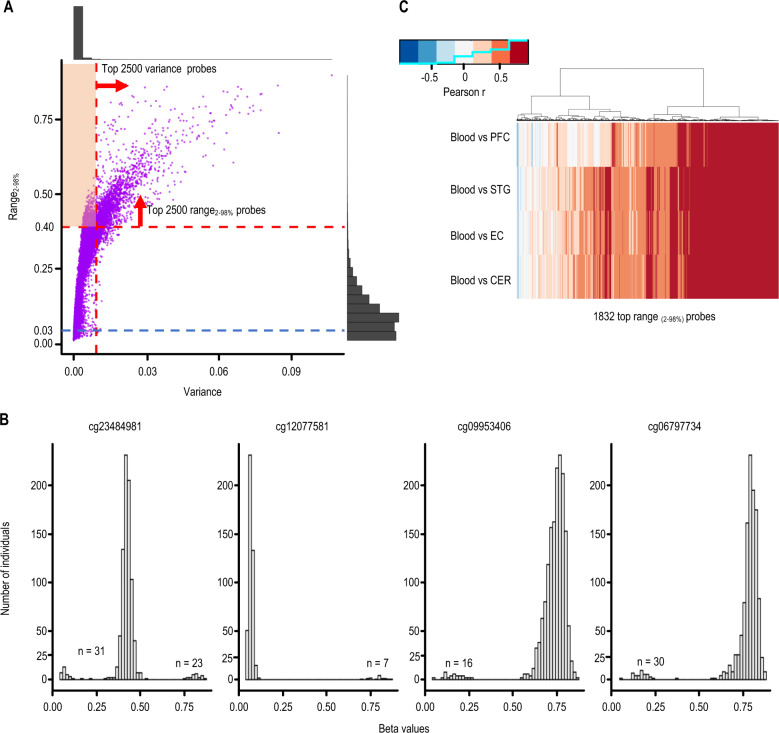
A new metric to assess interindividual variation in DNA methylation. **A** Range_2–98%_ vs. variance for each HM450 probe, across 847 samples in the training data set. Many probes (highlighted region) are in the top 2500 for range_2–98%_, but not for variance. **B** Distributions of individual-level beta values (proportional methylation) for four representative probes from the highlighted area in **A**. All show bimodal or trimodal distributions. Variance values for each of these four probes are 0.009, 0.008, 0.008, and 0.009, and range_2–98%_ values are 0.72, 0.56, 0.53, and 0.52, respectively. **C** Even after excluding those within CoRSIVs, the top 2500 probes by range_2–98__%_ generally show positive correlations between methylation in blood and the four brain regions, suggesting their utility for SZ case–control classification.

To determine whether our ability to classify SZ cases is due to SPLS-DA or the focus on CoRSIVs, we set out to develop a comparable classification model using the top 2500 most informative non-CoRSIV probes. We wished to select a set of non-CoRSIV probes which, like CoRSIVs, exhibit high interindividual variation. Because of the non-normal distribution of methylation at CoRSIVs, instead of variance, we assessed the inter-percentile range from the 2nd to 98th percentiles (which we term range_2–98%_) (Supplementary Methods, Supplementary Fig. S4 and Supplementary Table S2); 94.6% of the top range_2–98%_ probes detected in GSE84727 (whole-blood SZ case–control) are also classified as such in GSE80417 (whole-blood SZ case–control) (Supplementary Table S3). To illustrate the attributes of range_2–98__%_ we evaluated several probes among the top 2500 by range_2–98__%_ but not by variance (shaded region in Fig. 3A). In every instance, we observed bimodal or trimodal distributions (Fig. 3B), with a major mode separated from one or two minor modes. When we evaluated these high-range_2–98__%_ probes in the brain vs. blood data set [16] we found that, even after excluding those within CoRSIVs, most showed a substantial positive correlation in methylation between blood and four brain regions (Fig. 3C), comparable to the results observed for CoRSIVs (Fig. 1A). This was not the case for high-variance probes not classified as high range_2–98__%_ (Supplementary Methods and Fig. S5). An SPLS-DA risk classifier based on these high-variance probes alone had poor predictive power (Supplementary Fig. S6 and Table 2), indicating that the systemic nature of CoRSIVs is critical to the success of our classifier.

### Including additional covariates improves final model accuracy

**Fig. 4:**
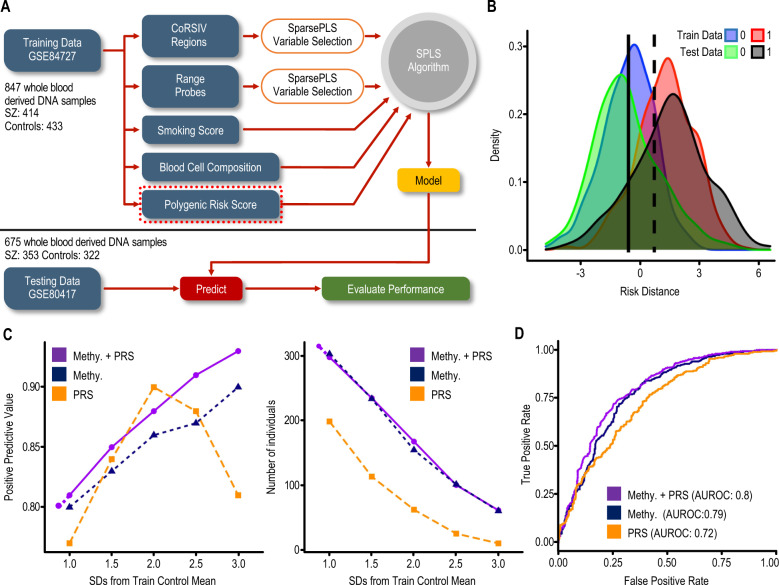
Final classification model incorporating DNA methylation at CoRSIVs and top range_2–98%_ probes, as well as blood cell composition, smoking score, and PRS. **A** Schematic diagram of the overall analytical approach. The feature selection and model building was done using SZ case–control HM450 data on 847 whole-blood DNA samples (GSE84727). Then, using the model, risk distances were calculated for an independent case–control set of 675 whole-blood DNA samples (GSE80417). **B** Risk-distance distribution in training and testing data. The solid vertical line shows the mean risk distance in training control samples, and the dashed line indicates 1SD above the mean of the training controls (0 = control, 1 = case). **C** Separate plots of PPV (left) and the number of individuals classified as cases (right) to evaluate classifier performance (as in Fig. 1D) for the final model including methylation and PRS, vs. models including either methylation or PRS. **D** AUROC curves of the models.

In addition to CoRSIV and top range_2–98__%_ probes, we included in the model variables assessing blood cell composition [27, 48], smoking [27], and genetic variation [5, 37, 49] (Fig. 4A). Consistent with the systemic nature of methylation at CoRSIVs, the SPLS-DA variable importance ranking (Supplementary Table S3) did not identify any leukocyte subtype as highly informative (i.e., within the top 10) in the model (Table 3). Smoking score, however, ranked as the most informative variable (Table 3 and Supplementary Table S3), consistent with the fact that individuals with SZ are more likely to smoke and smoke more heavily than controls [27]. Interestingly, the two genes associated with the top two model probes noted in Table 3 (*MYO1G* and *GFI1*) have previously been associated with SZ, although not at the same CpG sites [27]. The probes picked up by the final model showed a higher correlation between DNA methylation levels between blood and four brain regions (Supplementary Fig. S7), than probes previously identified as associated with SZ [27]. This final model consisted of 123 variables; importance scores are shown in Supplementary Table S3. The similarity between risk distance distributions in both the training and test sets (Fig. 4B) indicates the model performs well when classifying new data. We built separate SPLS-DA models with and without PRS as a covariate. The classification model built on PRS alone performed poorly, classifying only ~115 individuals as cases at 80% PPV (Fig. 4C). Surprisingly, including PRS in the methylation-based model did not substantially improve model performance; with or without PRS, just over 300 individuals were classified as cases, at 80% PPV (Fig. 4C, D and Table 2).

**Table 3:** Top 10 variables ranked by importance score in the final SPLS-DA model.

Top 10 variables in final model	Importance score	HM450 annotated gene
SmokingScore	0.58	–
cg12803068	0.39	*MYO1G*
cg12876356	0.37	*GFI1*
cg03751055	0.36	*MGMT*
cg06126421	0.30	*–*
cg09935388	0.29	*GFI1*
cg15135166	0.28	*PLEKHM2*
cg10540573	0.26	–
cg06791546	0.25	–
cg03680873	0.24	–

### The SZ classifier is not driven by excessive smoking of SZ cases

Smoking is both highly prevalent among SZ patients [50–52] and can affect DNA methylation in blood, raising additional potential for reverse causality. Previous EWAS studies [37, 49, 53] have identified 10 HM450 probes at which DNA methylation is strongly associated with current smoking. Because detailed smoking information was not available for each individual in the training and testing cohorts, a proxy variable (smoking score) was previously derived using DNA methylation values from these probes [27]. As described above, these known smoking-associated probes were excluded from our models at the outset. Nonetheless, to determine if smoking might somehow be driving our classifier we evaluated whether smoking score alone could predict SZ, but it was able to predict only 85 cases with 80% PPV (Table 2). To identify unknown smoking-associated CpGs that may be influencing our SZ classification model, we used a publicly available HM450 data set on whole blood of 464 individuals who were current, former, or never smokers [37]. Using the same CpGs identified by our SZ classification model, we built a binary classification model to classify smokers vs. non-smokers in this independent data set. This smoking classifier achieved an average AUROC of 0.69 in 10-fold cross-validation (CI: 0.67–0.77, Supplementary Fig. S8A), indicating our SZ classifier does include probes that are sensitive to smoking status. We therefore excluded the 10 probes most important for the smoking classifier (Supplementary Fig. S8B, C), as well as the smoking score, and re-trained the SZ classification model. This had a minimal impact; the model still classified 253 cases with 80% PPV (Table 2). Together, these analyses strongly indicate that our SZ model is not detecting differences in smoking between SZ cases and controls.

### Evaluating why inclusion of PRS did not improve the final model

**Fig. 5:**
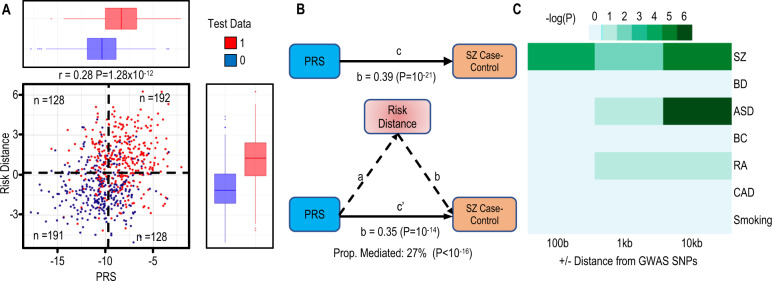
Evaluation of genetic influence on risk distance. **A** Plot of risk distances calculated from the final model (excluding PRS) vs. PRS for all individuals in the test set shows a weak positive correlation (Pearson *r* = 0.28, *P* = 1.28 × 10^−^^12^). The dashed horizontal and vertical lines show median risk distance and PRS, respectively (0 = control, 1 = case). **B** Mediational analysis indicates that 27% of the effect of PRS on disease status is mediated by CoRSIV methylation (i.e., risk distance). **C** Enrichment of GWAS SNPs identified for several conditions in the vicinity of CoRSIV probes in the classification model (SZ schizophrenia, BP bipolar disorder, ASD autism spectrum disorder, BC breast cancer, RA rheumatoid arthritis, CAD coronary artery disease, smoking). SNPs associated with SZ and ASD show stronger enrichment than those for non-neurological diseases.

Although the Pearson correlation between risk distance and PRS is only weakly positive (*r* = 0.28, *P* = 1.2 × 10^−12^), most individuals above the median risk distance are also above the 50th percentile for PRS (Fig. 5A). This correlation may explain why including PRS does not improve the model. On the other hand, many individuals with an intermediate PRS have elevated risk distance (Fig. 5A), suggesting an epigenetic predisposition not detected by PRS. Since genetic variants can influence methylation at CoRSIVs (methylation quantitative trait loci, mQTL) [20], we used mediational regression analysis to test whether the association between PRS and SZ case–control status may be mediated by CoRSIV methylation. Logistic regression showed a positive association between PRS and case–control status (*β* = 0.39, *P* = 1 × 10^−21^) (Fig. 5B). Including risk distance in the regression model (Fig. 5B) modestly reduced the effect size (*β* = 0.35, *P* = 1 × 10^−14^), indicating that risk distance (i.e., CoRSIV methylation) mediates 27% (*P* < 1 × 10^−16^) of the association between PRS and SZ case–control status. This partial mediation might reflect GWAS SNPs proximal to CoRSIVs wielding *cis* mQTL effects on CoRSIV methylation. Compared to other HM450 probes, CoRSIV and top range_2–98__%_ probes are 1.83-fold and 1.79-fold enriched for mQTL [54], respectively (see probe-level tabulation in Supplementary Table S4). And, indeed, analysis using the GWAS Catalog [55] showed robust enrichment for colocalization of model CpG probes and GWAS SNPs associated with SZ (Fig. 5C and Supplementary Methods).

## Discussion

To date, most case–control studies of DNA methylation in complex human diseases such as SZ have been conducted using Illumina HM450/EPIC arrays applied to whole-blood DNA. In general, however, the validity of extrapolating from whole blood to brain is unclear. Also, these studies were generally limited to detecting associations [26, 27, 56, 57], as opposed to empirically evaluating models to classify individuals based on the risk of SZ. Hence, our study represents both the first SZ case–control analysis of blood DNA methylation focused on systemic epigenetic variants and the first to apply the SPLS-DA machine-learning algorithm to DNA methylation data. Coupling these two innovations enabled an unprecedented ability to classify SZ cases and controls based on DNA methylation in blood.

Our initial attempt to train a supervised classification model (random forest, supplementary methods) using all CoRSIV regions performed poorly (AUROC of 0.67 in the independent test set). Our success, therefore, was in part due to the ability of SPLS-DA to include in the model only a small number of most informative variables (regularization). Also, the dimension reduction feature of SPLS-DA transformed the data from high-dimensional to low-dimensional space, facilitating 2-D projections that allowed us to calculate risk distance. In machine learning, classification accuracy can be improved by attempting to classify only those individuals for whom the model can make a reasonably accurate prediction [58]. So, to evaluate the performance of our model, we computed risk distances of individuals in the test set and classified individuals as cases, using various cutoffs.

Remarkably, our model based on blood methylation outperformed the model based on PRS, consistent with previous evidence [27, 59, 60] that interindividual epigenetic variation is an important etiologic factor in SZ. Considerable epigenetic variation is associated with genetics. Hence, it is not surprising to find a weak but significant correlation between risk distance and PRS. This suggests that genetic effects on SZ risk are, in part, mediated by mQTL effects at CoRSIVs. This interpretation is supported by our finding that GWAS SNPs associated with SZ are enriched in the vicinity of CoRSIVs in our final model. Despite evidence of shared GWAS loci between SZ and BP [61], we did not detect enrichments for BP GWAS SNPs. Significant enrichments were found for ASD; common genetic variants associated with both SZ and ASD have been reported [62]. Of four non-psychiatric diseases/conditions evaluated, only RA showed associations with GWAS variants, consistent with established links between RA and SZ [63, 64].

Given our contemporaneous design, the biggest caveat is the potential for reverse causality, which could occur, for example, if the methylation differences we are detecting are a consequence of antipsychotic medication or smoking. Unlike previous similar studies [26, 56, 57], however, we used two complementary approaches—applying our blood-based model to the brain, and testing for associations between risk distance, antipsychotic drug use, and smoking—to provide strong evidence against reverse causality. The highly significant enrichment of SZ GWAS SNPs in the vicinity of CpG probes identified by our model (Fig. 5C) and the finding that leukocyte subtype is not an important variable in our model (Table 3) are also inconsistent with simple confounding by medication effects on the blood methylation profile. Together, these findings suggest that the DNA methylation variants detected by our classifier were established prior to disease onset, and therefore may be used to assess the risk of SZ. A second limitation is that, due to the reliance on the HM450 array, our findings are based on only the 10% of known CoRSIVs that are informative on that platform [15, 20].

Our results indicate that by broadly assessing all known human CoRSIVs it may be possible to develop a highly accurate blood-based test to prospectively identify individuals at high risk for SZ. More generally, the approaches we describe serve as a proof of concept for the utility of CoRSIVs in personalized medicine, complementary to PRS. These innovations may ultimately enable blood-based epigenetic prediction models not only for SZ, but for a wide range of complex human diseases.

## Supplementary information

Supplementary Figures

Supplementary Methods

Supplementary Tables

## Data Availability

R source code developed for the analysis is available in Github and Zenodo [65].
