# Supplementary material for: A machine learning case–control classifier for schizophrenia based on DNA methylation in blood
Source: Transl Psychiatry. 2021 Aug 3;11:412. doi: 10.1038/s41398-021-01496-3 (PMC8329061; doi:10.1038/s41398-021-01496-3)
Supplement: Supplementary file 1 — Supplementary Figures [file 41398_2021_1496_MOESM1_ESM.pdf]

Supplementary Figures

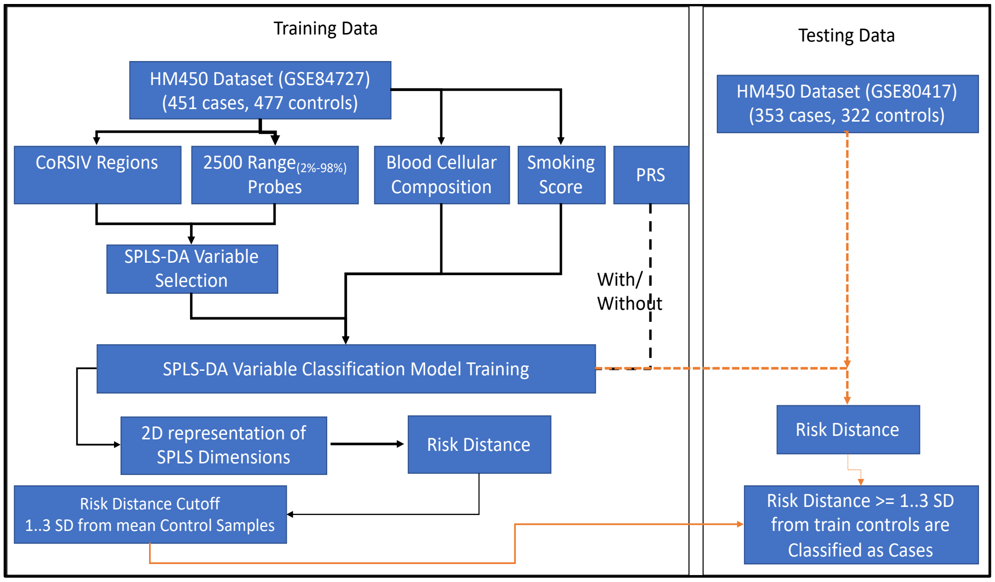

Fig. S1. Methodological overview of the analytical approach. SPLS-DA model parameter estimation and risk distance cutoff were derived using training data only. Testing data were used only to evaluate the model performance.

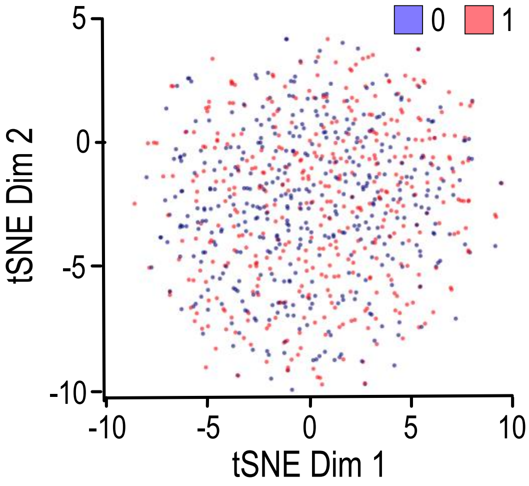

Fig. S2. tSNE 2D representation of HM450 CoRSIV probes shows no separation (0 = control, 1 = case).

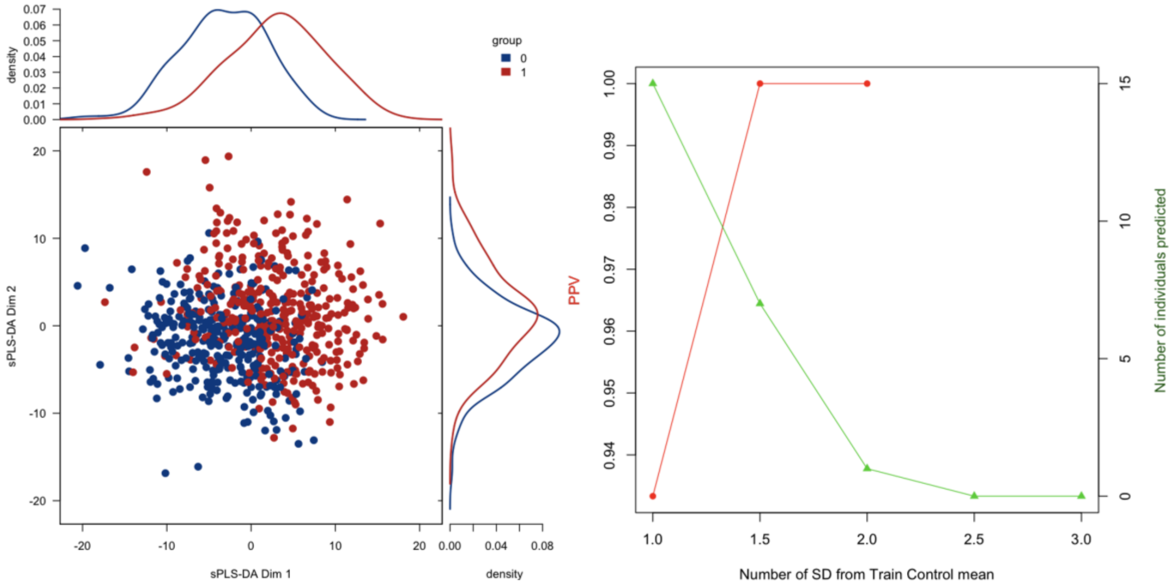

Fig. S3. SPLS-DA model built using Hannon et al. probes (SZ Case-Control DMPs). (Left) Scatter plot of 2-D coordinates of training data derived using SPLS-DA model trained utilizing the probes identified in Hannon et al 2016 (0 = control, 1 = case). Although the training data shows a separation, in the test data, cases were not generally classified as such by the model. Performance evaluation (right) shows only about 15 individuals showed high enough risk distance to be classified as cases.

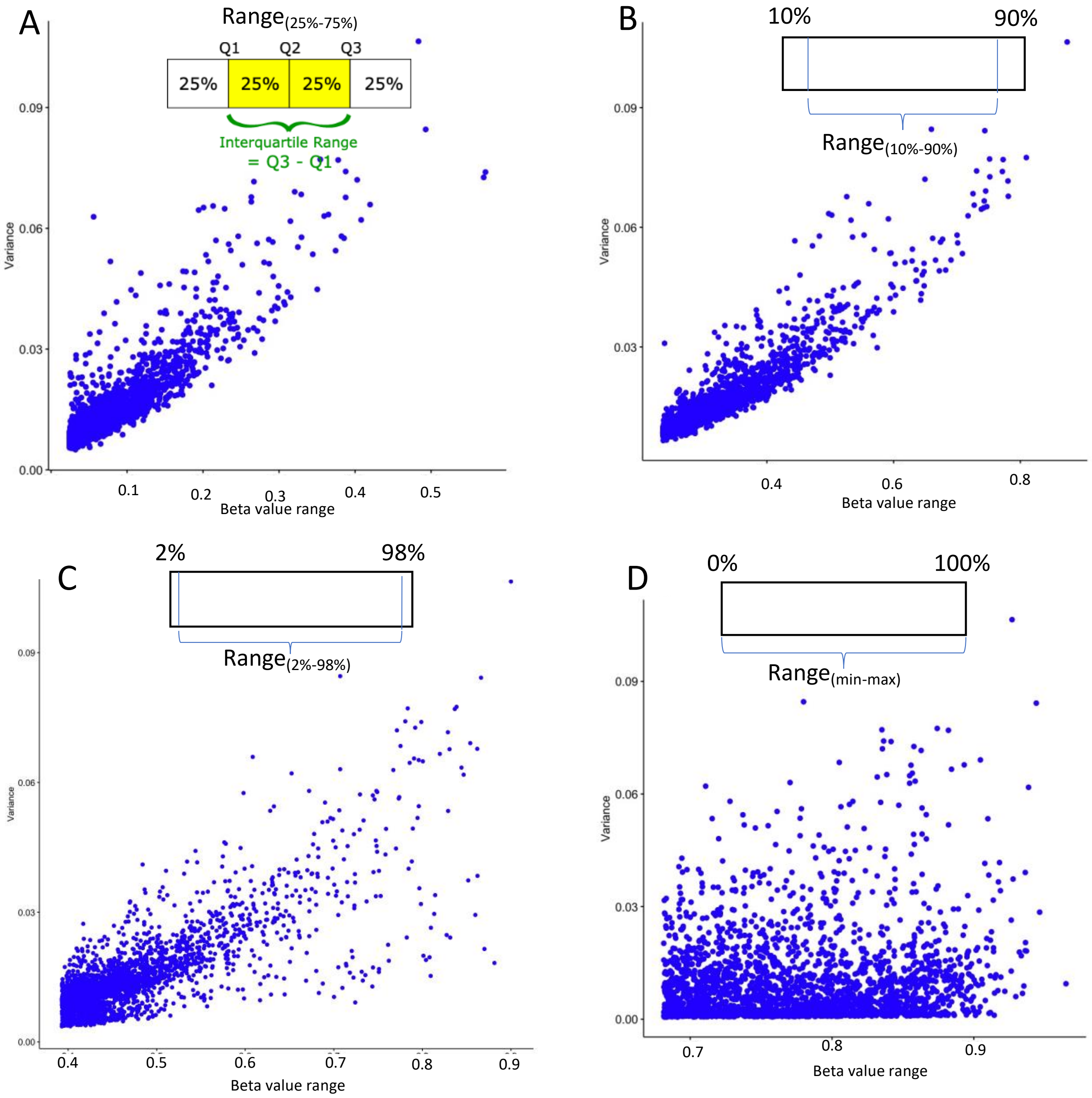

Fig. S4. Associations between beta value variance and various measures of beta value range for HM450 probes, across entire training set. Each panel shows the top 2500 probes when ranked from highest to lowest by different range types. **A.** Interquartile range, **B.** Inter-percentile range between 10% - 90%, **C.** Inter-percentile range between 2% - 98%, **D.** Full range between minimum and maximum beta values.

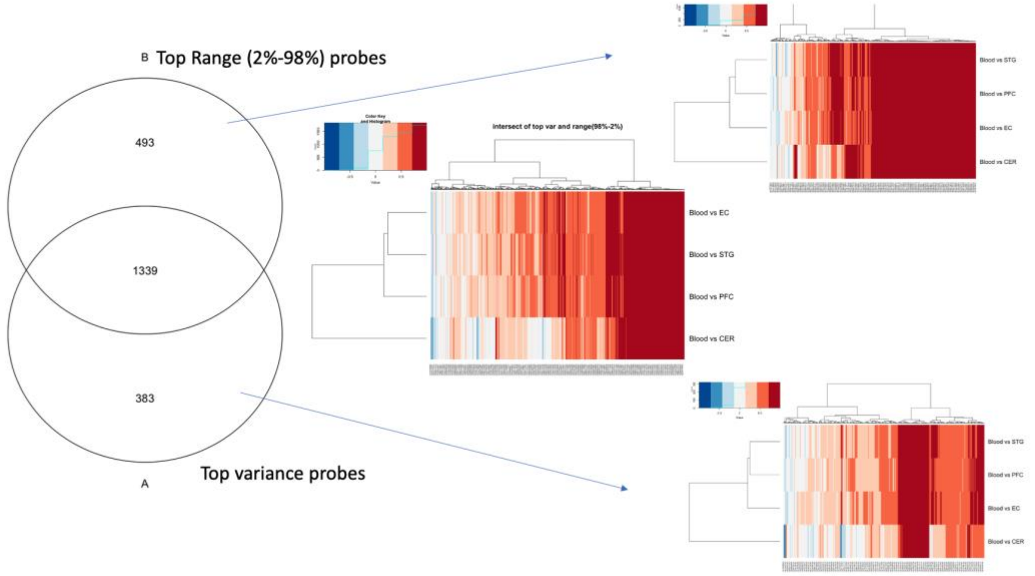

Fig. S5. Blood vs. four brain region correlations in top range$_{2\text{-}98\%}$ and top variance probes. A higher proportion of top range$_{2\text{-}98\%}$ probes (upper right) shows consistent positive correlation between blood and four brain regions compared to top variance probes, exclusive of both CoRSIV probes and range$_{2\text{-}98\%}$ probes (lower right). This indicates that range$_{2\text{-}98\%}$ captures a subset of HM450k probes that show correlated methylation levels between blood and these 4 brain regions.

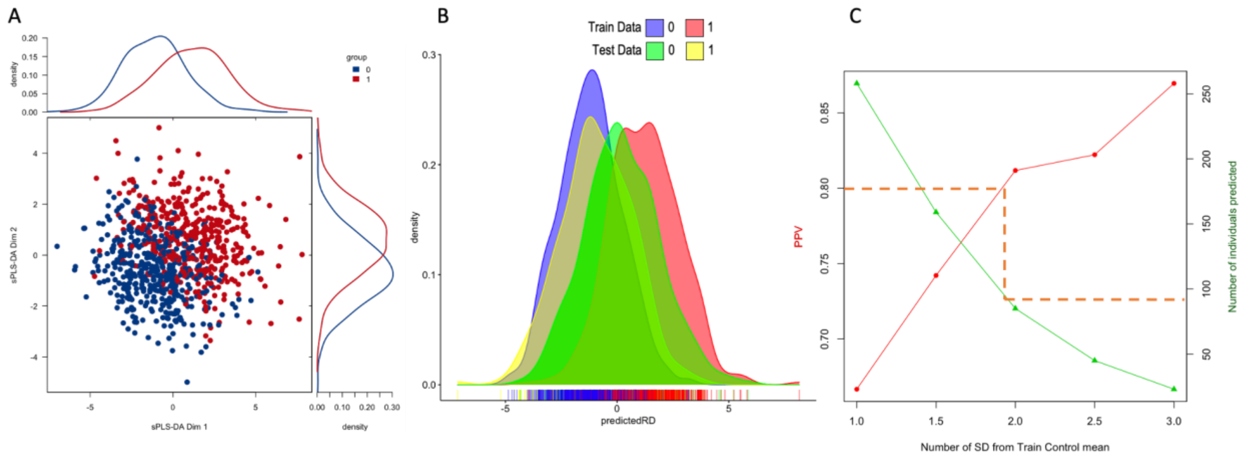

Fig. S6. SPLS-DA model performance when using top variance probes (excluding CoRSIVs). A. 2-D representation of training samples (0 = control, 1 = case). **B.** Distribution of risk distances for training and testing case-control samples (Red, Blue training case-controls and green and yellow test case-controls are represented). In the testing data, the mean risk distance of cases is *less than* that of controls. **C.** Performance evaluation; only about 80 individuals are classified as cases with 80% PPV.

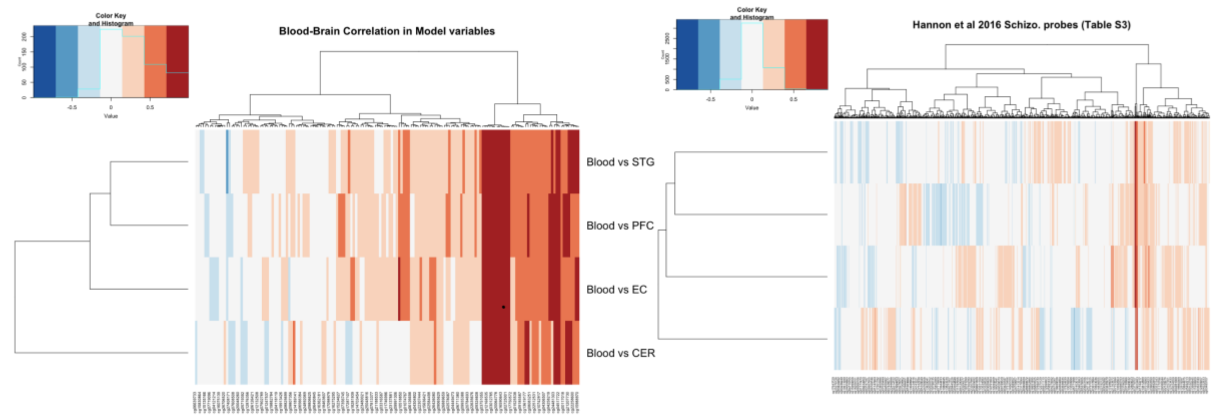

Fig. S7. Comparison of brain vs. blood correlations for probes in final model vs. those previously identify as differentially methylated in SZ vs. controls. **A.** The majority of probes in our final model show consistent positive correlation in blood and various brain regions (with the exception of cerebellum). **B.** By comparison, DMPs identified in Hannon et al 2016 only rarely show consistent positive cross-tissue correlation between blood and the 4 brain regions.

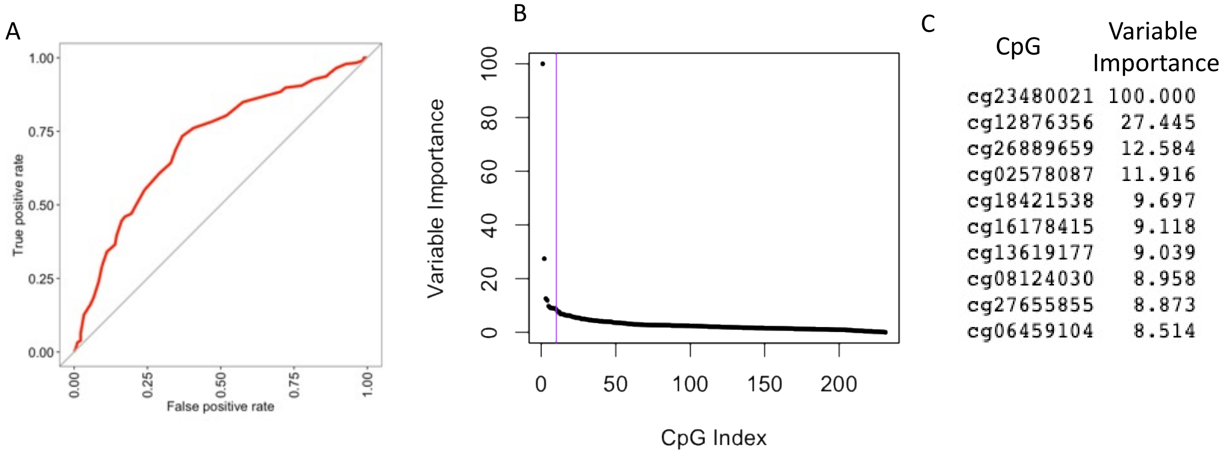

Fig. S8. Smoking Classification model using SZ model probes. A. The ROC curve for the binary classification of smoking vs non smoking individuals. **B.** The variable importance raning of the smoking classifier. The purple line shows the cut off for top 10 CpGs. **C.** The CpG ID and variable importance score of the top 10 CpGs.
