# Supplementary material for: A machine learning case–control classifier for schizophrenia based on DNA methylation in blood
Source: Transl Psychiatry. 2021 Aug 3;11:412. doi: 10.1038/s41398-021-01496-3 (PMC8329061; doi:10.1038/s41398-021-01496-3)
Supplement: Supplementary file 2 — Supplementary Methods [file 41398_2021_1496_MOESM2_ESM.docx]

Supplementary Methods

## Cohort Details

The machine learning model was trained on a publicly available SZ case-control Illumina HM450 dataset, GSE84727 (Aberdeen cohort). This training dataset contains 414 patients with SZ and 433 non-psychiatric controls who have self-identified as born in British Isles (95% in Scotland)^1^. The model was tested on an independent SZ case-control Illumina HM450 dataset, GSE80417 (University College London cohort). This testing dataset contains 353 patients with SZ and 322 non-psychiatric controls born in UK^2^. Data on the smoking score, blood cell composition estimates, and polygenic risk scores (PRS) for both training and testing data were calculated earlier^3^ and were used directly in this analysis without any modification. To demonstrate the systemic nature of the genomic regions focused in this study, Illumina HM450 DNA methylation data derived using matched DNA samples isolated from whole blood and 4 brain regions (prefrontal cortex, entorhinal cortex, superior temporal gyrus, and cerebellum) from 122 individuals were used (GSE59685)^4^. Another Illumina HM450 DNA methylation dataset derived from the prefrontal cortex (PFC) of 335 non-psychiatric controls across the lifespan and 191 patients with SZ (GSE74193) ^5^ were used to evaluate whether the model built on whole blood can classify SZ cases using PFC tissues. DNA methylation data (GSE50660) ^6^ from an epigenome-wide association study in peripheral-blood DNA in 464 individuals who were current, former and never-smokers were used to evaluate the smoking effect on the SZ classification model.

## CoRSIV Probes

In previous publications ^7-9^, we have identified human genomic regions (CoRSIVs) that show consistent DNA methylation across diverse tissues of the body. Of the ~480,000 probes on the HM450 array, only 3590 overlap known CoRSIVs ^10^. Since CpG sites within each CoRSIV are correlated, we averaged multiple probes within each CoRSIV, resulting in 1982 variables.

## Random Forest Classification Model

Using a supervised machine learning algorithm (Random Forest) to classify case-control samples based on CoRSIV methylation yielded poor classification; AUROC in the independent DNA methylation testing data, was only 67% . This could indicate that DNA methylation is associated with SZ at only a subset of CoRSIV probes. We identified SPLS-DA as a potentially effective machine learning method due to its simultaneous variable selection and dimension reduction capability^11,12^.

## SPLS-DA variable selection applied to CoRSIV regions

Case-Control dataset of CoRSIV regions is still a high-dimensional dataset with a large number of variables$\left( p \right)$ and a relatively small number of samples $\left( n \right)$. We therefore utilized SPLS-DA for improved classification by simultaneous variable selection and dimension reduction ^11,12^. The algorithm operates under the assumption that a relatively small fraction of the original variables are driving the underlying process and uses least absolute shrinkage and selection operator *(LASSO)* regularization ^13^ for variable selection, shrinking coefficients of unrelated variables to zero. We used the mixOmics R package ^14^ to apply SPLS-DA to the training dataset (GSE847272) and select top CoRSIV probes for consideration in the final classification model.

## SPLS-DA Variable selection applied to range_2%-98%_ probes

Due to the multi-modality of population-level DNA methylation data at CoRSIVs, in addition to variance, we evaluated range as a better measure of spread to extract probes with the greatest interindividual variation. Using the training dataset, we calculated the inter-percentile range between 2% and 98% (to exclude rare technical outliers) across 847 samples for CpG sites in the HM450 DNA methylation array. Across 847 individuals, for example, this approach excludes the lowest 16 and highest 16 values at each probe. For all HM450 probes, comparing variance and range_2-98%_ across the 847 individuals shows that the 2500 probes with the highest range_2-98%_ exhibit a minimum range_2-98%_ of 0.4 Beta (40% methylation) whereas, the 2500 highest variance probes include some with an interindividual range_2-98%_ of just 0.03 Beta (3% methylation – blue dashed line). Of the top 2500 probes by variance, only 1339 are among the top 2500 by range_2-98%_.Then we extracted the top 2500 probes with the highest inter-percentile methylation range, which we term ‘range_(2%-98%)_’. We used 2500 as the cutoff since there is an overlap of 455 probes between CoRSIV probes and top range probes. Once these are removed, each probe set has approximately 2000 unique probes. SPLS-DA variable selection was applied to the range probes using training data, and selected variables were identified as important for discriminating SZ cases from controls.

## Building the final classification model using the SPLS-DA algorithm on the training data

The final SPLS-DA classification model was built using the selected CoRSIV probes, selected range probes, blood-cell composition, smoking scores, and including (or not) PRS^3^. Parameter tuning was done using training data with 10-fold cross-validation for optimal components and variables for the model. The reduced dimension of the training data can be represented in a 2-dimensional plot with case-control labeling. The 2-D plot can be used to visualize the separation of the case-control data and calculate the risk distance.

## Calculating Risk Distance for Training and Testing Data

Based on the training data case-control separation, a direction vector can be identified as $\mathrm{var}\left( dim1 \right)i+var\left( dim2 \right)j$*.* Along this vector, Euclidian Distance from the origin (0,0) to all training data points can be calculated. For every sample in the independent testing set, 2-D coordinates can be calculated using SPLS-DA model parameters. Then along the same unit vector identified in the training data, Risk Distances are computed from the origin (0,0) to new testing samples.

## Model Performance Evaluation

In machine learning, classification accuracy can be improved by attempting to classify only those individuals for whom the model can make a reasonably accurate prediction^15^. The distribution of Risk Distances for each training and testing sample can be represented in a 1-D density plot for easy comparison to observe the difference between cases and controls. Based on the training data, positive risk distance implies a higher risk for SZ. Model performance is evaluated by setting cutoffs at various risk distance standard deviation multiples (1, 1.5, 2, 2.5, 3) above the control mean risk distance, to classify individuals as SZ cases. Positive predictive value (PPV) is the probability that subjects with a positive screening test truly have the disease. To compare among models, we standardized at 80% PPV; at various standard deviations above the control mean, we tallied the number of individuals classified as cases at 80% PPV.

## Association with psychiatric medication

The OPCRIT database includes medication data on 423 cases of the training set. Cases with missing/incomplete information about antipsychotic drug use were removed, leaving 232 individuals for this analysis. To evaluate the effect of medication use on risk distance, we calculated the Spearman correlation between risk distance and chlorpromazine equivalent doses (a standardized quantitative method for comparing dosages of different drugs).

## Overlap with GWAS variants

We used SNP locations from the GWAS Catalog^16^ to test for enrichment of GWAS SNPs associated with SZ, bipolar disorder (BP), and autism spectrum disorder (ASD) in the vicinity of probes in the final SPLS-DA model. We additionally considered some non-psychiatric diseases included in the GWAS catalog: breast cancer (BC), rheumatoid arthritis (RA), and coronary artery disease (CAD)^17^. Fisher exact tests were calculated for the enrichment of model probes within 100, 1,000, or 10,000 bp of these disease GWAS SNPs.

## References:

1. International Schizophrenia, C. Rare chromosomal deletions and duplications increase risk of schizophrenia. *Nature* **455**, 237-41 (2008).

2. Datta, S.R. *et al.* A threonine to isoleucine missense mutation in the pericentriolar material 1 gene is strongly associated with schizophrenia. *Mol Psychiatry* **15**, 615-28 (2010).

3. Hannon, E. *et al.* An integrated genetic-epigenetic analysis of schizophrenia: evidence for co-localization of genetic associations and differential DNA methylation. *Genome Biol* **17**, 176 (2016).

4. Lunnon, K. *et al.* Methylomic profiling implicates cortical deregulation of ANK1 in Alzheimer's disease. *Nat Neurosci* **17**, 1164-70 (2014).

5. Jaffe, A.E. *et al.* Mapping DNA methylation across development, genotype and schizophrenia in the human frontal cortex. *Nat Neurosci* **19**, 40-7 (2016).

6. Tsaprouni, L.G. *et al.* Cigarette smoking reduces DNA methylation levels at multiple genomic loci but the effect is partially reversible upon cessation. *Epigenetics* **9**, 1382-96 (2014).

7. Gunasekara, C.J. *et al.* A genomic atlas of systemic interindividual epigenetic variation in humans. *Genome Biol* **20**, 105 (2019).

8. Van Baak, T.E. *et al.* Epigenetic supersimilarity of monozygotic twin pairs. *Genome Biol* **19**, 2 (2018).

9. Silver, M.J. *et al.* Independent genomewide screens identify the tumor suppressor VTRNA2-1 as a human epiallele responsive to periconceptional environment. *Genome Biol* **16**, 118 (2015).

10. Gunasekara, C.J. & Waterland, R.A. A new era for epigenetic epidemiology. *Epigenomics* **11**, 1647-1649 (2019).

11. Chun, H. & Keles, S. Sparse partial least squares regression for simultaneous dimension reduction and variable selection. *J R Stat Soc Series B Stat Methodol* **72**, 3-25 (2010).

12. Chung, D. & Keles, S. Sparse partial least squares classification for high dimensional data. *Stat Appl Genet Mol Biol* **9**, Article17 (2010).

13. Tibshirani, R. Regression Shrinkage and Selection Via the Lasso. *Journal of the Royal Statistical Society: Series B (Methodological)* **58**, 267-288 (1996).

14. Rohart, F., Gautier, B., Singh, A. & Le Cao, K.A. mixOmics: An R package for 'omics feature selection and multiple data integration. *PLoS Comput Biol* **13**, e1005752 (2017).

15. Haw-Shiuan Chang, E.L.-M., Andrew McCallum. Active Bias: Training More Accurate NeuralNetworks by Emphasizing High Variance Samples. (2017).

16. Buniello, A. *et al.* The NHGRI-EBI GWAS Catalog of published genome-wide association studies, targeted arrays and summary statistics 2019. *Nucleic Acids Res* **47**, D1005-D1012 (2019).

17. Chan, R.F. *et al.* Independent Methylome-Wide Association Studies of Schizophrenia Detect Consistent Case-Control Differences. *Schizophr Bull* **46**, 319-327 (2020).
